# Na^+^-stimulated phosphate uptake system in *Synechocystis *sp. PCC 6803 with Pst1 as a main transporter

**DOI:** 10.1186/1471-2180-11-225

**Published:** 2011-10-11

**Authors:** Surachet Burut-Archanai, Julian J Eaton-Rye, Aran Incharoensakdi

**Affiliations:** 1 Laboratory of Cyanobacterial Biotechnology, Department of Biochemistry, Faculty of Science, Chulalongkorn University, Bangkok 10330, Thailand; 2 Department of Biochemistry, University of Otago, P.O. Box 56, Dunedin, New Zealand; 3 Center of Excellence for Marine Biotechnology, National Center for Genetic Engineering and Biotechnology, Pathumthani 12120, Thailand

## Abstract

**Background:**

Most living cells uptake phosphate, an indispensable nutrient for growth from their natural environment. In *Synechocystis *sp. PCC 6803, the cells lack phosphate-inorganic transport (Pit) system but contain two phosphate-specific transport (Pst) systems, Pst1 and Pst2. We investigated the kinetics of Pi uptake of these two Pst systems by constructing the two mutants, ΔPst1 and ΔPst2, and comparing their kinetic properties with those of the wild-type cells under both Pi-sufficient and deficient conditions. The effects of pH and Na^+ ^on the uptake of phosphate in *Synechocystis *were also studied.

**Results:**

Growth rates of the two mutants and wild type were similar either under phosphate-sufficient or deficient condition. The *K_m _*for phosphate uptake was 6.09 μM in wild type and this was reduced to 0.13 μM in ΔPst1 cells and 5.16 μM in the ΔPst2 strain. The *V_max _*values of 2.48, 0.22, and 2.17 μmol • (min • mg of chlorophyll *a*)^-1 ^were obtained for wild type, the ΔPst1 and ΔPst2 strains, respectively. A monophasic phosphate uptake was observed in wild-type cells. The uptake of phosphate was energy and pH-dependent with a broad pH optimum between pH 7-10. Osmolality imposed by NaCl stimulated phosphate uptake whereas that imposed by sorbitol decreased uptake, suggesting stimulation of uptake was dependent upon ionic effects.

**Conclusion:**

The data demonstrate that Pst2 system of *Synechocystis *has higher affinity toward phosphate with lower *V_max _*than Pst1 system. The Pst1 system had similar *K_m _*and *V_max _*values to those of the wild type suggesting that Pst1 is the main phosphate transporter in *Synechocystis *sp. PCC 6803. The *K_m _*of Pst1 of *Synechocystis *is closer to that of Pit system than to that of the Pst system of *E. coli*, suggesting that *Synechocystis *Pst1 is rather a medium/low affinity transporter whereas Pst2 is a high affinity transporter.

## Background

Phosphorus is an essential mineral nutrient for all organisms, for example, for the biosynthesis of nucleotides such as ATP as well as DNA and RNA, and for the functional regulation of proteins by phosphorylation. However, inorganic phosphate (Pi), the only form of phosphorus that can be directly utilized by cells, is often limiting in natural environments where it is frequently present at nanomolar levels [1]. In response to Pi limitation, the expression of genes for proteins that participate in the uptake and/or in the scavenging of Pi is induced under the control of a Pi-specific two-component system [2-5]. In *Escherichia coli*, Pi uptake is carried out by two kinetically distinct systems: the high affinity phosphate-specific transport (Pst) system and the low affinity phosphate-inorganic transport (Pit) system, with Michaelis-Menten (*K_m_*) values about 0.25 μM and 20 μM, respectively [6]. In *E. coli*, the high-affinity Pst system belongs to the Pho regulon and when environmental Pi is in excess (greater than 4 μM) the expression of genes of the Pho regulon is not induced [5]. Therefore under Pi-replete conditions Pi uptake occurs via the Pit system.

Many cyanobacteria also exhibited different kinetic parameters for Pi uptake when grown under Pi-limiting conditions than when grown under Pi-replete conditions [7,8]. For example, *Synechococcus *sp. PCC 7942 exhibits a lower *K_m _*for Pi uptake when grown under Pi-limiting conditions. This organism contains both low-affinity and high-affinity Pi transport systems where the high-affinity Pi transport activity is regulated by the periplasmic Pi-binding protein SphX [8]. In contrast, a low affinity Pit-like Pi transport system is thought to be absent in *Synechocystis *sp. PCC 6803 (hereafter *Synechocystis *6803) [9]. This cyanobacterium was previously shown to contain two Pst systems, Pst1 and Pst2, that are up-regulated in response to Pi limitation [4]. It is well known that the growth of cyanobacteria relies both on the size of the pool of internal polyphosphate and on their ability to take up Pi from the natural environment with fluctuating Pi levels [10,11]. It is therefore of interest to investigate the uptake of Pi by Pst1 and Pst2 of *Synechocystis *6803. In this study we determined the kinetics of each Pst system using deletion mutants of each system in *Synechocystis *6803. We demonstrated that Pst1 was the main Pi transporter whereas Pst2 might play a role in the uptake of Pi under low Pi environments.

## Results

### Growth of wild type and mutants

**Figure 1:**
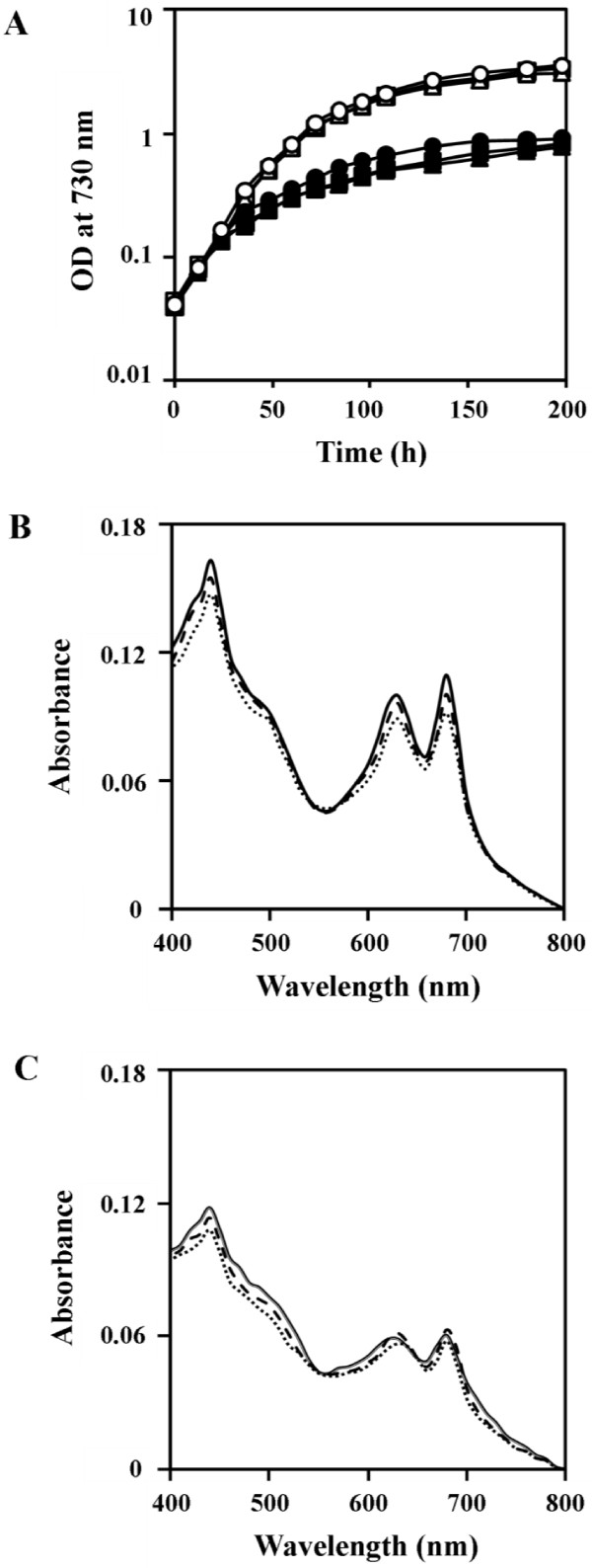
**Photoautotrophic growth and absorption spectra of *Synechocystis *sp. PCC 6803 wild type, and the ΔPst1 and ΔPst2 mutants**. (A) Photoautotrophic growth of strains as measured by the optical density at 730 nm in BG-11 (open symbols) or Pi-limiting BG-11 (closed symbols); wild type (circles); ΔPst1 (triangles); and ΔPst2 (squares). Absorption spectra of whole cells after 3 days in BG-11 of wild type (solid lines), ΔPst1 (dot lines) and ΔPst2 (dash lines) under Pi-sufficient conditions (B) and Pi-limiting conditions (C).

The growth of wild-type *Synechocystis *6803 was similar to that of the mutants lacking either Pst1 (ΔPst1 cells) or Pst2 (ΔPst2 cells) in BG-11 medium (Figure 1A). Under Pi-limiting conditions, the three strains also showed similar growth characteristics during the first two days but later on showed slightly slower growth rates than in BG-11 medium. The analysis of total Pi content of all three strains showed a small increase of total Pi during the first 24 h under Pi-replete conditions (Table 1). At 96 h, total Pi content decreased slightly or remained stable. On the other hand, under Pi-limiting conditions the three strains showed a decrease of total Pi at 24 h and only marginal contents were detected later in growth. In both situations, the total Pi content of the three strains was very similar at all time points tested.

**Table 1:** Pi contents of three strains of *Synechocystis *sp

Strain	Total cellular Pi (pmol cell^-1^)			
	
	0 h	24 h	48 h	96 h
Wild type	0.23	0.25	0.22	0.21
ΔPst1	0.21	0.22	0.20	0.21
ΔPst2	0.21	0.24	0.20	0.17

PCC 6803 grown in BG-11 under Pi-replete conditions for various times

The absorption spectra showed no difference among the three strains when grown in BG-11 (Figure 1B). Likewise, similar spectra were obtained for all strains grown under Pi-limiting conditions with the peaks at 440 nm and 680 nm, corresponding to chlorophyll *a*, and the peak at 620 nm, corresponding to phycobilins, all being reduced (Figure 1C).

### Phosphate uptake

**Figure 2:**
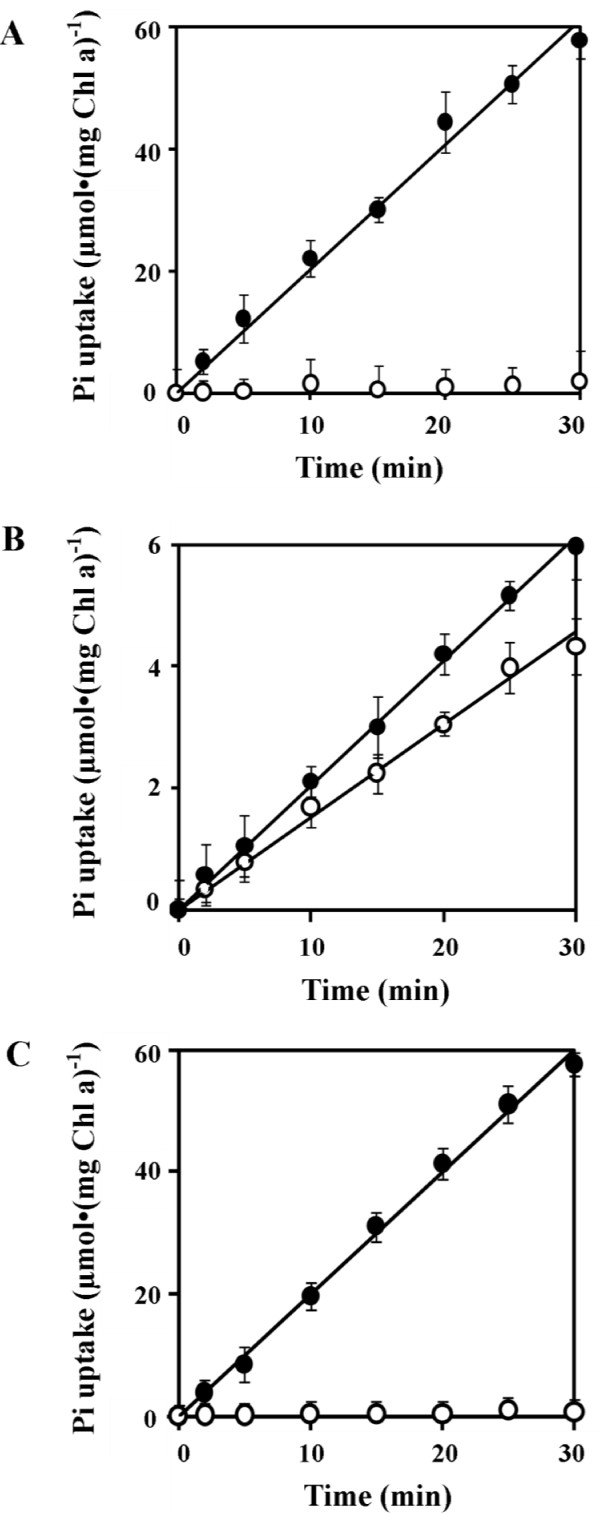
**Phosphate uptake of cells grown in BG-11 (open circles) or Pi-limiting BG-11 for 24 h (closed circles) of wild type (A), ΔPst1 (B) and ΔPst2 (C)**. The concentrations of Pi used in the assay were 50 μM for all three strains. Note the difference in the scale on Y-axis for Figure 2B.

One-day Pi-starved *Synechocystis *6803 cells showed a linear increase in Pi uptake during 30 min whereas no apparent uptake was observed in cells under Pi-replete conditions (Figure 2A). However, the ΔPst1 mutant showed Pi uptake under Pi-limiting and Pi-replete conditions (Figure 2B), but these Pi uptake activities by ΔPst1 cells accounted for only ~10% of that observed for wild-type cells under Pi-limiting conditions.. In contrast, the ΔPst2 mutant showed similar rates of Pi uptake to that of wild type (Figure 2C).

**Figure 3:**
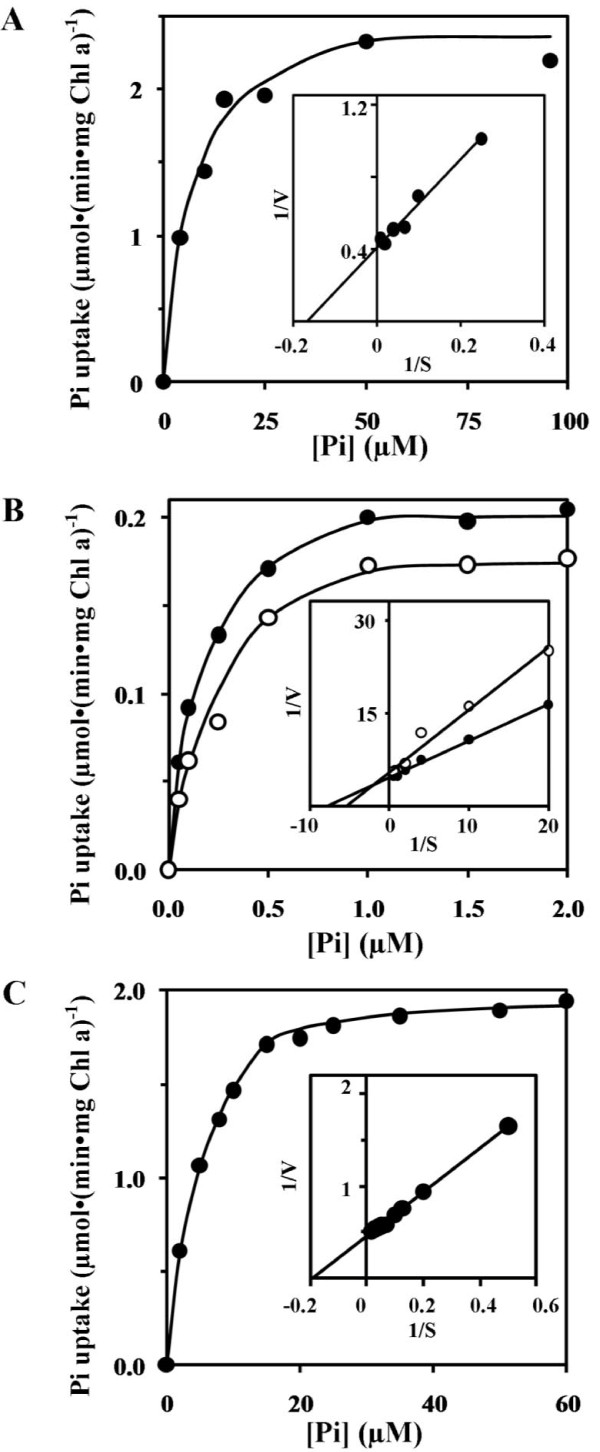
**Kinetics of phosphate uptake by strains grown in BG-11 (open circles) or Pi-limiting-BG-11 (closed circles) for 24 h: wild type (A), ΔPst1 (B) and ΔPst2 (C)**. Inset represents a double-reciprocal plot of the concentration dependence of the initial rates of Pi uptake. The units on the X- and Y- axis are μM^-1 ^and (min • mg Chl a) • μmol^-1^, respectively.

All strains showed saturation kinetics for the uptake of Pi (Figure 3A-C). Under Pi-limiting conditions, double-reciprocal plots yielded a *K_m _*of 6.09 and 5.16 μM and maximum velocity (*V_max_*) of 2.48 and 2.17 μmol • (min • mg chlorophyll *a*)^-1 ^for wild type and the ΔPst2 mutant, respectively (Figure 3A, C insets). The kinetic parameters for both wild type and the ΔPst2 strains under Pi-replete conditions could not be obtained due to their very low uptake capacity. The Pi uptake of the ΔPst1 mutant either under Pi-sufficient or Pi-limiting conditions appeared to be saturated at very low concentration of Pi with a *K_m _*of 0.13 and 0.18 μM and *V_max _*of 0.22 and 0.18 μmol • (min • mg chlorophyll *a*)^-1 ^under Pi-limiting and Pi-sufficient conditions, respectively (Figure 3B).

### Effect of light/dark and metabolic inhibitors on phosphate uptake

We demonstrated that the Pi uptake activity of wild-type cells was inhibited in the presence of various metabolic inhibitors and under dark incubation (Table 2). Under dark incubation, the presence of the photosystem II-specific inhibitor 3-(3, 4-dichlorophenyl)-1, 1-dimethylurea and KCN, led to an ~50% reduction of Pi uptake. Moreover, uptake was significantly decreased in the presence of ion-gradient dissipating agents such as, gramicidin, the sodium ionophore, amiloride and valinomycin. Strong inhibition was also caused by carbonyl cyanide *m*-chlorophenylhydrazone with the remaining activity ~ 25%. The Pi uptake was also diminished by N-ethylmaleimide. Altogether, these results indicated that the uptake of Pi by *Synechocystis *6803 is energy-dependent and that an ion gradient is necessary for the uptake.

**Table 2:** Effect of metabolic inhibitors, phosphate analogs, and incubation in the dark on phosphate uptake in *Synechocystis *sp. PCC 6803^a^

Treatment	Phosphate uptake (%)
Control	100 ± 2
NaF 1 mM	93 ± 5
N, N-dicyclohexylcarbodiimide 40 μM^b^	91 ± 6
Na^+ ^ionophore 10 μM	91 ± 4
Gramicidin10 μM	80 ± 3
Amiloride 20 μM	77 ± 5
Valinomycin 20 μM	77 ± 4
Monensin 20 μM	69 ± 4
KCN 5 mM	54 ± 3
3-(3, 4-dichlorophenyl)-1, 1-dimethylurea 20 μM^b^	51 ± 6
Dark	48 ± 5
N-ethylmaleimide 1 mM	31 ± 6
Carbonyl cyanide *m*-chlorophenylhydrazone 40 μM^b^	23 ± 6

^a^Cells were preincubated with inhibitors for 30 min before the addition of K_2_HPO_4 _to initiate uptake. Data are the mean of three experiments ± SD. ^b^Cells were preincubated with inhibitors for 2 min before assays.

### Effect of external pH on phosphate uptake

**Figure 4:**
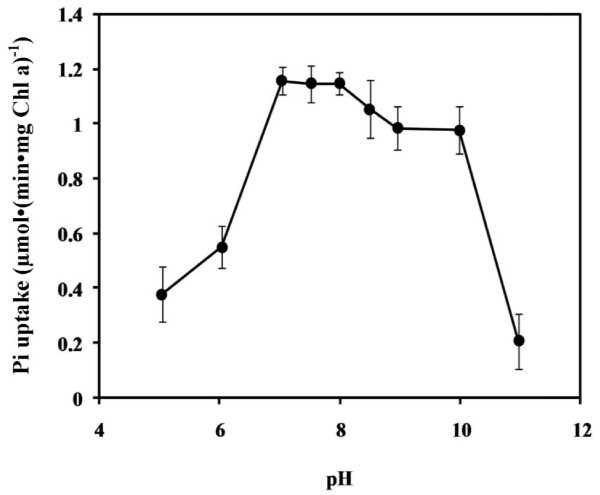
**Effect of external pH on the initial rates of phosphate uptake in *Synechocystis *sp. PCC 6803**. The 24 h cells grown in Pi-limiting medium were washed and resuspended in 25 mM each of MES/KOH (pH 5.0-6.0), HEPES/KOH (pH 7.0-8.5), and ethanolamine/KOH (pH 10.0-11.0) After 2 h incubation, aliquots were taken for assays of Pi uptake.

The Pi uptake ability of wild-type cells was tested at different pH ranging from pH 5 to 11 using 25 mM of either MES/KOH (pH 5.0-6.0) or HEPES/KOH (pH 7.0-8.5) or ethanolamine/KOH (pH 10.0-11.0). The *Synechocystis *6803 cells exhibited similar Pi uptake activity under broad alkaline conditions ranging from pH 7 to 10 (Figure 4).

### Effect of osmolality on phosphate uptake

**Figure 5:**
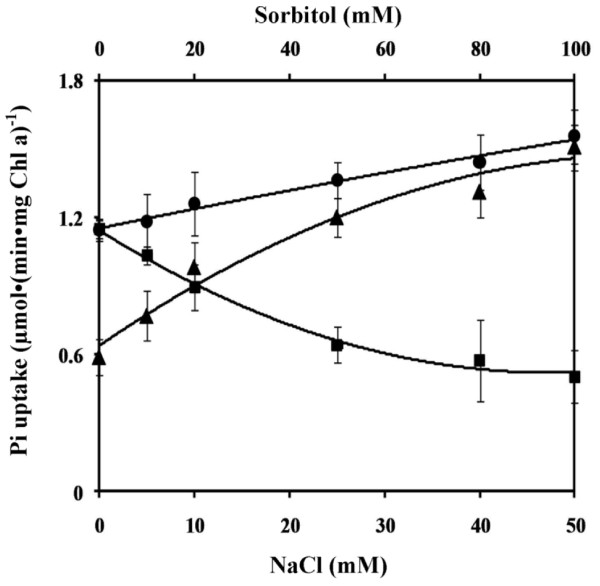
**Effect of salt stress and osmolality stress on the initial rates of phosphate uptake in *Synechocystis *sp. PCC 6803**. The 24 h cells grown in Pi-limiting medium were washed and resuspended in 25 mM HEPES/KOH buffer pH 7.5 containing NaCl (circles), NaCl and sorbitol to keep osmolality equivalent to 100 mOsm • kg^-1 ^(triangles), and sorbitol (squares). After 2 h incubation, aliquots were taken for assays of Pi uptake.

The Pi uptake in many cyanobacteria was shown to be strongly activated by the addition of Na^+ ^[12]. The presence of NaCl could generate ionic stress and osmotic stress. To test whether ionic stress or osmotic stress affected Pi uptake, experiments were carried out in the presence of various concentrations of NaCl and sorbitol or a combination of both with a fixed osmolality equivalent to 100 mOsmol • kg^-1^. Figure 5 shows that NaCl stimulated Pi uptake whereas sorbitol reduced Pi uptake. The osmolality of 100 mOsmol • kg^-1 ^contributed solely by sorbitol caused about 50% reduction in Pi uptake. However, increasing the concentration of NaCl while keeping the osmolality at 100 mOsmol • kg^-1 ^led to a progressive increase of Pi uptake.

## Discussion

The *pst1 *and *pst2 *operons belonging to the Pho regulon in *Synechocystis *6803 were shown to be both up-regulated when cells grown in BG-11 (containing 175 μM Pi) were transferred to a Pi-free medium [3,4,13]. These conditions have routinely been used to investigate the Pho regulon in cyanobacteria [2,14,15]. *Synechocystis *6803 cells are able to survive under Pi-limiting conditions following initial growth in BG-11 although photoautotrophic growth and pigment content decreased [3]. Similarly, the absence of either the Pst1 or Pst2 Pi-uptake system did not prevent growth, suggesting that the mutants had sufficient Pi stored over the course of the measurement [16]. This was partly substantiated by the analysis of total Pi which showed similar Pi content among wild type, ΔPst1 and ΔPst2 strains up to 96 h growth in both Pi-limiting and Pi-replete conditions.

Our kinetics studies showed that Pi uptake characteristic of Pst1 (ΔPst2 strain) was similar to that of wild type whereas Pi uptake by Pst2 (ΔPst1 strain) accounted for about 10% of the wild type (Figure 3). This suggested that Pst1 is the main Pi transporter of *Synechocystis *6803. Pst2 of *Synechocystis *6803 contributed very weakly for the uptake of Pi despite its higher affinity than that of Pst1 system. The Pst2 transporter was taking up Pi with similar kinetics when grown either under Pi-limiting or Pi-replete conditions (Figure 2B). This suggested that the expression of Pst2 was constitutive whereas that of Pst1 was inducible by Pi-limitation (Figure 2C). The Pst2 system might be important when *Synechocystis *cells encounter Pi-poor environments. Under these environments the absence of Pst2 might lead to a severe internal Pi shortage leading to a strong induction of the expression of the Pst1 system. The cells can then take up Pi at a higher rate to sustain growth under Pi-poor environments. On the other hand, even in the presence of Pst2 (as in the case for wild type), internal Pi shortage might also occur since the Pi uptake capacity of Pst2 was relatively low. Since the contribution to the uptake of Pi by Pst2 is rather low, the uptake of Pi in *Synechocystis *relies mainly on Pst1 which is considered as a medium/low affinity transporter in comparison to the high affinity transporter of Pst1 system in *E. coli*. These observations suggest that *E. coli *might adjust and survive better than *Synechocystis *under low Pi environments. It is likely that some relations exist between the usual Pi concentration of a biotope and the *K_m _*of the Pi uptake system of the microorganisms thriving in this biotope.

Some evolutionary adaptation might exist between Pst1 and Pst2 in *Synechocystis *6803 which is advantageous for its survival in environments with fluctuating Pi availability. In this respect, it is worth mentioning that the analysis using BLASTP [17] revealed a low % similarity of amino acid sequences of periplasmic Pi-binding proteins belonging to Pst1 and Pst2 systems (37% to 57%). In contrast, both the transmembrane permease subunits and the cytosolic ATP-binding subunits of these Pst1 and Pst2 systems shared high % similarity of amino acid sequences spanning from 67% to 84%. This suggested that differences in kinetic properties between Pst1 and Pst2 are accounted for mainly by differences in the periplasmic Pi-binding protein subunits.

The uptake of Pi in response to changes in external pH by *Synechocystis *6803 was similar to that by *Synechococcus *sp. PCC 7942 [18]. Both cyanobacteria had poor uptake activity at acidic pH. At external pH of 7 which is lower than the pK_2 _of phosphoric acid the monovalent species (H_2_PO_4_^-^) predominates whereas at external pH of 10 almost all Pi is in the divalent form (HPO_4_^2-^) [19]. The fact that there were no significant differences in Pi uptake at pH 7 and 10 (Figure 4) suggested that the Pi uptake system in *Synechocystis *6803 can recognize both H_2_PO_4_^- ^and HPO_4_^2-^. The ability of *Synechocystis *6803 to bind two different Pi species is advantageous to its survival especially under fluctuating external pH and low Pi availability.

The increased Pi uptake activity by NaCl is ascribed to an ionic rather than an osmotic effect since an osmotic stress of the same strength achieved with a non-ionic sorbitol caused a reduction in Pi uptake (Figure 5). It is possible that the presence of Na^+ ^might facilitate the uptake of Pi, as in *E. coli *where it is transported as neutral metal phosphate [20]. The driving force for the uptake of Pi in *Synechocystis *6803 is likely to be ATP generated by ion gradient or ion gradient itself. Indeed, the effect of the inhibitors tested on this uptake support this hypothesis. The fact that Pi uptake is Na^+^-stimulated and that the uptake is favorable at alkaline pH can support this contention.

## Conclusion

*Synechocystis *cells can survive under Pi-limiting conditions following initial growth in BG-11 medium. The uptake of Pi in *Synechocystis *6803 is accomplished mainly by Pst1 despite its lower affinity for Pi than that of Pst2. The expression of Pst2 might be useful when cells encounter low Pi environments. Pi uptake is stimulated by alkaline pH as well as by ionic solute such as NaCl whereas it is inhibited by non-ionic solute (sorbitol) generating osmotic stress.

## Methods

### Strains and growth conditions

Axenic cells of *Synechocystis *6803 were grown photoautotrophically in BG-11 medium at 30°C under continuous illumination (warm white fluorescent tubes) at 25 μE m^-2 ^s^-1^, with continuous shaking on a rotary shaker (Innova™ 4340, New Brunswick Scientific, USA) at 160 rpm. For Pi-limiting experiments, Pi was replaced by an equimolar solution of KCl [3]. The Pst1 and Pst2, Pi transport systems of *Synechocystis *6803 were deleted by replacing the *pst1 *and *pst2 *operons with a spectinomycin-resistance cassette and kanamycin-resistance cassette, respectively, as described previously [14]. The growth rate was monitored by measuring the optical density at 730 nm. The Pi contents of wild type, ΔPst1 and ΔPst2 strains were determined according to Shi et al. [21].

### Assay of phosphate uptake

Cells grown in BG-11 medium for 3 days were washed twice by centrifugation and resuspension in Pi-limiting BG-11 medium. The washed cells were subsequently grown in either BG-11 or Pi-limiting BG-11 medium for 24 h before being washed twice by centrifugation and resuspension in Pi-free buffer to an optical density at 730 nm of 0.3. The uptake experiment was initiated by the addition of K_2_HPO_4 _solution at room temperature. At different time intervals, aliquots were withdrawn, filtered through a 0.45 μm membrane filter and the remaining Pi in the filtrate was determined by the colorimetric method [22].

## Authors' contributions

AI and JER conceived the project, designed the experiments, provided advice, and wrote the manuscript. SB designed and performed the experiments, prepared tables and figures, and partially wrote the manuscript. All authors read and approved the final manuscript.
